# Environmental Stimuli and Phytohormones in Anthocyanin Biosynthesis: A Comprehensive Review

**DOI:** 10.3390/ijms242216415

**Published:** 2023-11-16

**Authors:** Lei Shi, Xing Li, Ying Fu, Changjiang Li

**Affiliations:** State Key Laboratory of Plant Environmental Resilience, College of Biological Sciences, China Agricultural University, Beijing 100193, China; 18811065839@163.com (L.S.); m18438561106@163.com (X.L.); yingfu@cau.edu.cn (Y.F.)

**Keywords:** anthocyanin, environmental stress, phytohormone, MBW complex

## Abstract

Anthocyanin accumulation in plants plays important roles in plant growth and development, as well as the response to environmental stresses. Anthocyanins have antioxidant properties and play an important role in maintaining the reactive oxygen species (ROS) homeostasis in plant cells. Furthermore, anthocyanins also act as a “sunscreen”, reducing the damage caused by ultraviolet radiation under high-light conditions. The biosynthesis of anthocyanin in plants is mainly regulated by an MYB-bHLH-WD40 (MBW) complex. In recent years, many new regulators in different signals involved in anthocyanin biosynthesis were identified. This review focuses on the regulation network mediated by different environmental factors (such as light, salinity, drought, and cold stresses) and phytohormones (such as jasmonate, abscisic acid, salicylic acid, ethylene, brassinosteroid, strigolactone, cytokinin, and auxin). We also discuss the potential application value of anthocyanin in agriculture, horticulture, and the food industry.

## 1. Introduction

Anthocyanins are a group of flavonoid pigments in plants that produce purple, pink, red, or blue colors, and play important roles in regulating plant development, growth, and the interactions between the plant and the environment [1,2,3,4,5,6,7]. For example, the accumulation of anthocyanins in plants can attract pollinators and seed distributors and can help the plant defend itself against UV-B stress, salinity, drought, and cold stress [8,9,10]. Furthermore, anthocyanin accumulation is associated with fruit veraison and ripening in many crop and fruit plants in parallel with the activation of anthocyanin-synthesis-related enzymes, such as in grape (*Vitis vinifera* L.), and contributes as an important nutrient for human beings [11,12,13,14,15,16].

**Figure 1:**
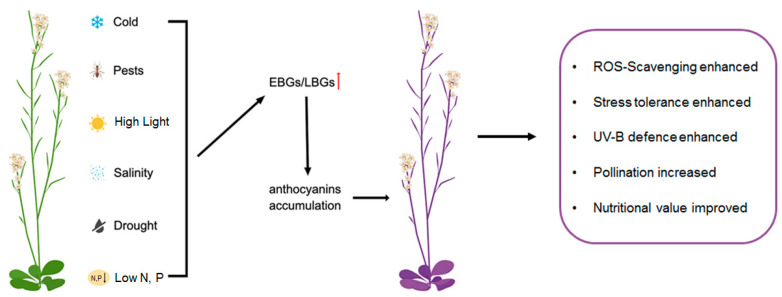
Plants accumulate anthocyanins in response to biological or abiotic stresses for better growth. When exposed to stresses, the expressions of anthocyanin biosynthetic genes (EBGs/LBGs) are upregulated, subsequently leading to an increase in anthocyanin accumulation within the plants. This heightened accumulation assists the plants in defending against stress by means of scavenging excessive reactive oxygen species (ROS) and reallocating nitrogen resources, among other mechanisms. Red arrow indicates up-regulation of EBGs or LBGs.

The biosynthesis of anthocyanin in plants is mainly controlled by some anthocyanin biosynthetic structural genes, which are divided into two groups: early biosynthetic genes (EBGs, such as *CHS*, *CHI*, and *F3′H*) and late biosynthetic genes (LBGs, such as *DFR*, *LDOX*, *UF3GT*, *UGT75C1*, and *3AT1*) [7,17,18,19]. The EBGs encode the key enzymes to synthesize the precursors common to flavonoids or other phenolics, while LBGs encode the enzymes specifically committed to anthocyanins [18] (Figure 1). For more comprehensive reviews of these enzymes’ functions and the basic biosynthetic pathway of anthocyanins, we refer readers to other reviews [2,18,20,21,22,23].

In plants, the expression of LBGs is mainly controlled by an MYB-bHLH-WD40 (MBW) complex, which consists of MYB transcriptional factors (TFs) (such as GL1, PAP1/MYB75, VvMYBA1, and VvMYBA2), bHLH TFs (such as TT8, GL3, and EGL3), and WD40 protein (such as TTG1) in *Arabidopsis* and grape (*Vitis vinifera* L.) [24,25,26]. In recent years, more and more members of the MBW complex in different plants have been identified, and different environmental stimuli or plant hormone signaling pathways can regulate the expression of MBW members, or they can regulate the assembly and the activity of MBW complex to control the anthocyanin biosynthetic gene expressions [18]. To date, many new regulators in different signals involved in MBW-mediated anthocyanin biosynthesis have been identified (Table 1).

In this review, we focus on the regulation network controlling anthocyanin biosynthesis, which is influenced by environmental stimuli and plant hormones. We also delve into the recent discoveries of key regulatory factors within the anthocyanin biosynthesis pathways in plants. Additionally, we underscore the potential applications of anthocyanins in crop breeding.

## 2. Environmental Stimuli and Anthocyanins

### 2.1. Light and Anthocyanin Biosynthesis in Plants

Light is crucial in regulating anthocyanin biosynthesis in plants [53]. Without light, the anthocyanin biosynthesis in plants is nearly blocked [27,54,55]. In *Arabidopsis*, ELONGATED HYPOCOTYL 5 (HY5) and its coactivators BBX20/21/22 play key roles during light-induced anthocyanin biosynthesis [17,56,57]. *hy5* and *bbx20 21 22* mutants accumulate lower anthocyanin levels compared with wild-type plants [56,58]. HY5 is a key component in light signaling and was shown to act as a transcriptional activator of *CHS*, *CHI*, *F3′H*, *MYB12*, and *PAP1/MYB75* via its direct binding to ACEs (ACGT-containing elements), leading to the accumulation of anthocyanins in response to visible and UV-B light [17,59,60,61,62]. In contrast, VvBBX44 directly represses VvHY5 and VvMYBA1 to balance the anthocyanin concentration under light in grape (*Vitis vinifera* L.) [28]. Studies showed that in etiolated seedlings, CONSTITUTIVE PHOTOMORPHOGENIC1 (COP1), which is a RING-finger E3 ubiquitin ligase, promotes the polyubiquitination and subsequent degradation of HY5 and HYH (HY5 homolog) in *Arabidopsis*, which indicates that COP1 acts as a negative regulator of light-induced anthocyanin biosynthesis [17,59,63]. Furthermore, studies also showed that COP1 can directly target PAP1 and PAP2 and promote their degradation in the absence of light [27]. When exposed to light, COP1 is inhibited by activated photoreceptors, such as CRYPTOCHROMEs (CRYs), PHYTOCHROMEs (PHYs), and UV RESISTANCE LOCUS 8 (UVR8), thereby allowing for the accumulation of positively acting TFs [53,64].

**Figure 2:**
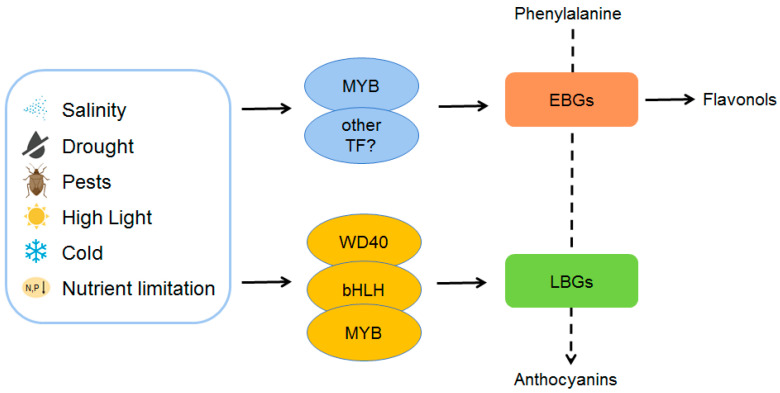
Mechanism of environmental-stress-related anthocyanin biosynthesis. Environmental stresses can promote anthocyanin biosynthesis by inducing the expressions of anthocyanin biosynthesis regulatory genes (such as *MYBs*, *WD40*, and *bHLHs*). Black arrows indicate positive regulation. This model is modified from Araguirang et al. (2022) [53].

Under high-light conditions, genes involved in anthocyanin synthesis are significantly induced, leading to the accumulation of anthocyanins in plants [29,53,65,66]. For instance, high light induces more expressions of *MYB112* and *PAP1*/*MYB75*, therefore leading to more anthocyanin structural gene expressions [29,65]. It is reported that the MAPK pathway plays an important role in high-light-induced anthocyanin biosynthesis. MAP KINASE4 (MPK4) could be activated in response to light and phosphorylate PAP1/MYB75 to increase the stability of PAP1/MYB75, which is essential for light-induced anthocyanin accumulation [65]. On the other hand, the class II HD-ZIP protein HAT1 negatively regulates the high-light-induced anthocyanin accumulation through competitively interacting with MYB75 and interferes with the formation of the MBW complex, thereby repressing the LBG (such as *DFR*, *LDOX*, and *UF3GT*) expressions via recruiting histone deacetylase mediated by TOPLESS (TPL) [30]. There is a report indicating that under high light treatment, the light attenuation function of anthocyanins is more important than their antioxidant role in photoprotection [66] (Figure 2).

### 2.2. Salinity Induces Anthocyanin Biosynthesis

Salinity is one of the most widespread abiotic stresses all over the world, and it can induce secondary stress in plants, such as osmotic stress, iron stress, and oxidative stress [67,68]. It was reported that salt stress leads to the accumulation of anthocyanins, which are proposed to be antioxidants that scavenge excessive ROS induced by salinity [8,9,29,31,33,69,70]. *pap1-D* plants have increased anthocyanin accumulation and radical scavenging activity [71], and they exhibit an enhanced tolerance to high salinity [8]. Truong et al. (2018) reported that enhanced anthocyanin biosynthesis leads to better growth performance of plants under low-nitrate and high-salinity conditions via the regulation of nitrate metabolism [72]. Moreover, this group found that increasing the amount of anthocyanins by knocking out *FLS1* in *pap1-D* mutant could improve the salt stress tolerance under high NO_3_^-^ application [73]. Moreover, ectopic expression of *AtDFR* leads to a high level of anthocyanin accumulation and confers significant salinity tolerance in *Brassica napus* L. [70]. Furthermore, *Arabidopsis* UDP-glycosyltransferases UGT79B2 and UGT79B3 were shown to improve salt tolerance via the modulation of anthocyanin accumulation [9].

Salinity induces the expressions of many anthocyanin-biosynthesis-related genes, including both EBGs (*CHS*, *CHI*, *F3′H*) and LBGs (*DFR*, *LDOX*, *UF3GT*, *UGT75C1*, *3AT1*, etc.) in plants [29,33]. To date, several transcriptional regulators are identified as key factors that regulate salt-induced structural genes. PAP1/MYB75 is the most known TF that upregulates salt-induced anthocyanins, where the salt-induced anthocyanins could be enhanced by adding sucrose, which further indicates that salt signaling may engage in cross-talk with sucrose signaling [8,33]. AtMYB112 is another TF that positively regulates salinity-induced anthocyanins. Salt stress (150 mM NaCl) can induce the expression level of *AtMYB112* and lead to the upregulation of downstream genes (*AtMYB7* and *AtMYB32*), hence promoting salt-induced anthocyanin biosynthesis in *Arabidopsis* [29]. Recently, research into *Arabidopsis* indicates that the adaptor protein EAR motif-Containing Adaptor Protein (ECAP) interacts with PAP1/MYB75 and represses its activity in normal conditions, while different levels of salinity can remove the ECAP’s repression of the PAP1/MYB75-dependent MBW complex by both jasmonate (JA) signaling and an unknown pathway (Figure 2) [33].

### 2.3. Drought Promotes Anthocyanin Accumulation in Plants

Drought is one of the most common environmental stresses that plants suffer [74,75,76,77]. Lots of studies found that drought promotes anthocyanin accumulation in plants, and the accumulated anthocyanins serve as an important antioxidant to scavenge ROS induced by drought stress [9,78]. Recently, there was a study showing that the ectopic overexpression of *StAN1* (a key TF that regulates *ANS*, *DFR*, and *UFGT* from *Solanum tuberosum*) in tobacco plants leads to the overaccumulation of anthocyanins, and the transgenic plants have a stronger drought tolerance compared with wild-type plants [79] (Figure 2). A study of *Arabidopsis* indicated that different abiotic-stress-induced anthocyanins have different localizations at the tissue and organ levels [80]. ECAP was shown to mediate the drought-induced anthocyanin accumulation in *Arabidopsis* [31]; however, whether this process is dependent on JA signaling remains unclear.

### 2.4. Cold Induces Anthocyanins Accumulation in Plants

Cold stress, which is a common abiotic factor, exerts significant impacts on plant growth, development, and overall fitness [81]. Plants have evolved complex molecular responses to counteract the detrimental effects of cold stress, and one intriguing aspect of this response is the induction of anthocyanin synthesis [4,82]. Cold stress triggers the upregulation of specific transcription factors, such as MYBs (such as BrMYB2 and AtMYB75) and bHLHs (such as BrTT8), which bind to the promoter regions of anthocyanin biosynthetic genes (such as *BrDFR1*, *BrANS1*, and *BrUF3GT2*), initiating the transcriptional cascade that leads to anthocyanin production [83] (Figure 2). Anthocyanins possess potent antioxidant properties to scavenge ROS generated during cold stress [84]. ROS accumulation can result in cellular damage, membrane disruption, and oxidative stress. By effectively neutralizing ROS, anthocyanins contribute to the maintenance of cellular integrity and homeostasis, reducing the potential for cold-induced damage [84,85]. In addition to ROS scavenging, anthocyanins may also play a role in photoprotection [86]. Cold stress often leads to photoinhibition due to the deregulation of photosynthesis homeostasis. Anthocyanins can act as “sunscreen” pigments, absorbing excess light energy and dissipating it as heat, thereby protecting the photosynthetic apparatus from photodamage [87,88]. In apple, MdMYB308L serves as a positive regulator of cold tolerance and anthocyanin accumulation through its interaction with MdbHLH33 and undergoes protein degradation mediated by MdMIEL1, highlighting the pivotal role of dynamic MYB-bHLH protein complexes in plant growth and development regulation [34]. In grape (*Vitis vinifera* L.), anthocyanin accumulation in leaves induced by the low temperature in autumn can help to enhance their cold tolerance [89].

### 2.5. Anthocyanins Confer Pest and Disease Resistance in Plants

Recent studies suggest that anthocyanins may contribute to plant defense against pests and diseases [4,10,90,91,92] (Figure 2). For instance, the content of anthocyanins was significantly increased in rust-infected symptomatic tissue of *Malus* apple, and the anthocyanin biosynthetic genes *McDFR* and *McLOX* were also upregulated [91]. Another study showed that some flavonoid glycosides in *Basella alba* could inhibit the growth of *Spodoptera litura* larvae [92]. However, the molecular mechanisms of pest- or disease-mediating anthocyanins accumulation still need further investigation.

### 2.6. Nutrient-Limitation-Induced Anthocyanin Accumulation in Plants

Low phosphorus and low nitrogen stresses often induce the accumulation of anthocyanins in plants. Under conditions of phosphorus and nitrogen limitation, plants adapt to environmental stress by modulating nutrient allocation and metabolic pathways, including increasing the synthesis of anthocyanins [35,36,93] (Figure 2). This phenomenon is known as nutrient-limitation-induced anthocyanin accumulation [35,94,95]. This represents a physiological response strategy of plants to environmental stress that is aimed at enhancing their resilience.

Phosphorus is a vital component in energy transfer and molecular signaling, and thus, its scarcity prompts plants to allocate resources strategically. In response, plants regulate anthocyanin biosynthesis as a part of a larger mechanism to enhance their adaptive fitness [36,96]. Under low-phosphorus conditions, increased expression of MYB transcription factors, such as PHOSPHATE STARVATION RESPONSE 1 (PHR1), was observed [39,40,97]. PHR1 can initiate a signaling cascade that directly upregulates anthocyanin-related genes, such as *F3′H* and *LDOX* in *Arabidopsis* [40]. Furthermore, SPX4 also controls the PAP1 protein level and affects the PAP1-mediated anthocyanin pathway under low-phosphorus stress [39]. Notably, the Gibberellin (GA)-DELLA signaling pathway also regulates the phosphate-starvation-induced anthocyanin in *Arabidopsis* [36].

Similarly, low-nitrogen stress triggers a sophisticated interplay of molecular mechanisms that culminate in the induction of anthocyanin accumulation. Nitrogen, as an essential component of amino acids and proteins, plays a central role in plant growth and development. In response to nitrogen scarcity, plants redistribute resources to favor metabolic pathways that improve nutrient efficiency [98,99]. This reallocation often coincides with the accumulation of anthocyanins, as observed in the model plant *Arabidopsis thaliana*. *NLA* (*nitrogen limitation adaptation*) plays a key role in regulating N-limitation-induced anthocyanin synthesis. The *nla* mutant cannot accumulate anthocyanins and instead produces an N-limitation-induced early senescence phenotype [35]. The MYB TFs also play a key role in low-N-induced anthocyanin accumulation, and the PAP1 loss-of-function mutant showed low anthocyanin accumulation and low survival rate under a low-N stress treatment [93,100]. Furthermore, a *DFR*-deficient mutant *tt3* also showed significantly lower survival rates after N starvation compared with the wild type in *Arabidopsis* (Figure 2). These studies all indicate that low-N-induced anthocyanin accumulation plays a substantial role in plant tolerance to low-N stress. Moreover, studies also showed that the GA-DELLA module is involved in nitrogen-deficiency-induced anthocyanin accumulation. DELLAs could interact with PAP1 and enhance the transcriptional activity of PAP1 to promote the expressions of *F3′H* and *DFR* [37,101].

## 3. Plant Hormones and Anthocyanins

### 3.1. Strigolactone Promotes Anthocyanin Accumulation

**Figure 3:**
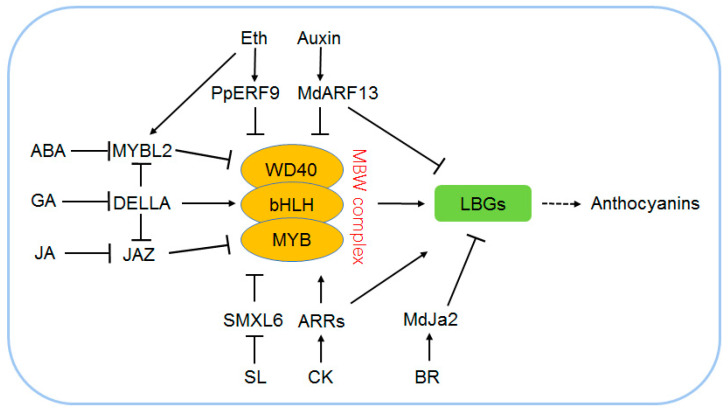
Mechanism of plant-hormone-related anthocyanin biosynthesis in *Arabidopsis*, apple, and pear. The major regulation network between MBW complex and plant hormones (such as Auxin, ABA, JA, GA, and BR) shows that plant hormones can promote or repress anthocyanin biosynthesis via positively or negatively regulating MBW complex or directly regulating LBG expressions. Black arrows indicate positive regulation and perpendicular lines indicate negative regulation.

Strigolactone (SL) is an important phytohormone that participates in regulating shoot branching, leaf shape, and metabolism in plants [41]. A report indicates that SL can promote the accumulation of anthocyanin, which further confers adaption to a low-phosphate condition in *Arabidopsis* [102]. Wang et al. 2020 found that the SL-mediated anthocyanin biosynthesis was triggered by the degradation of SMXL6, SMXL7, and SMXL8 proteins through the 26S proteasome pathway, further promoting the expressions of *PAP1*/*PAP2* in *Arabidopsis*. The SMXLs are the key repressors and have dual functions in SL signaling. On one hand, SMXL6 can directly bind the promoters of *SMXL6*/*7*/*8* and negatively regulate their transcription; on the other hand, SMXL6 can function as a transcriptional repressor to inhibit the expressions of SL-responsive genes, including *PAP1*/*PAP2* [41] (Figure 3). However, further investigation is still needed to determine which TFs directly bind to SMXLs and function upstream of *PAP1*/*PAP2*.

### 3.2. JA Mediates Anthocyanin Biosynthesis

JA is a plant hormone that participates in plant defense against biotic/abiotic stress and regulates plant metabolisms [18,103]. JA was shown to have a positive effect on anthocyanin biosynthesis in plants [32,42,104]. Studies showed that multiple members of JA signaling in plants are involved in anthocyanin biosynthesis. The mutants of JA receptor gene *CORONATINE INSENSITIVE1* (*COI1*) and JA biosynthetic gene *12-oxophytodienoate reductase 3* (*OPR3*) show a low anthocyanin phenotype compared with wild-type *Arabidopsis* seedlings [31,32,33,42]; while the key repressors of JA signaling, namely, JAZs, are negative regulators of anthocyanin accumulation [31,32,105], and ECAP acts as an adaptor protein mediating the interacting of JAZ6/8 and co-repressor TPR2 to form the JET complex and plays a negative role in anthocyanin biosynthesis in *Arabidopsis* [31]. It has become clear that JA regulates anthocyanin biosynthesis by affecting the stability and activity of the MBW complex [31,32]. JAZ1/8/11 can competitively inhibit the formation of the MBW complex and hence inhibit the expressions of LBGs [32]. ECAP-mediated repression is mainly achieved via histone deacetylation on the target genes of the MBW complex rather than by competitively binding with MBW members [31], while JA promotes the degradation of JAZs and allows for the activity of the MBW complex, thereby leading to the upregulation of LBGs [31,32]. In apple, a study showed that the JAZ1-TRB1-MYB9 complex dynamically modulates the JA-mediated accumulation of both anthocyanin and proanthocyanidin [43] (Figure 3). Furthermore, in grape (*Vitis vinifera* L.), methyl jasmonate (MeJA) treatment promotes anthocyanin accumulation by regulating a VvmIR156-VvSPL9 module in the early stage of color conversion [44].

### 3.3. Abscisic Acid Promotes Anthocyanin Accumulation

Abscisic acid (ABA) is a plant hormone that regulates plant growth, development, and stress response [106,107]. There are also many reports indicating that ABA mediates development-dependent anthocyanin biosynthesis in the leaves and fruits of many plants, such as apple, strawberry, sweet berry, bilberry, and lycium plants [45,108,109,110,111,112]. The key TF of ABA signaling in apple, namely, MdABI5, was found to positively regulate ABA-induced anthocyanin biosynthesis by directly upregulating the expression of *MdbHLH3* and interacting with MdbHLH3 to enhance the MdMYB1–MdbHLH3 interaction, which led to the upregulation of *MdDFR* and *MdUF3GT* transcripts [45]. Elongators also play important roles in regulating ABA signaling and anthocyanin biosynthesis. The Elongator subunit (ELO1/ELP4, ELP2, and ELP6) mutants in *Arabidopsis* are hypersensitive to ABA and accumulate more anthocyanins than the wild type [46]. Further investigation shows that Elongator positively regulates the expression of MYBL2, which is a negative regulator of the MBW complex [46] (Figure 3).

### 3.4. Salicylic Acid Mediates Anthocyanin Biosynthesis

Salicylic acid (SA) is a vital plant hormone that regulates immunity against biotrophic and semi-biotrophic pathogens [113]. SA also positively regulates anthocyanin accumulation in many plants, such as grape, pomegranate, and *Arabidopsis* [48,114,115]. It shows that knocking out the SA receptor NPR1 leads to a low-anthocyanin-content phenotype in *Arabidopsis* compared with the wild type, regardless of the airborne fungus treatment [48]. Furthermore, the MBW complex also participates in airborne-fungus-induced anthocyanin biosynthesis [48]. This indicates that SA signaling mediates airborne-fungus-induced anthocyanin accumulation, though the key components of the TFs or other co-regulators are still not very clear.

### 3.5. Brassinosteroid and Anthocyanin Biosynthesis

Brassinosteroid (BR) is a new class of plant hormone that participates in many physiological processes, including anthocyanin biosynthesis, in many plants [49,116,117,118]. In grape, the exogenous application of BR and its analogs led to an increase in anthocyanin accumulation in its fruit [119]. However, a study of apple showed that BR treatment inhibits the synthesis of anthocyanin, and the MdBZR1-MdJa2 module plays a negative role in the control of downstream LBG expressions [49] (Figure 3). Furthermore, studies of *Arabidopsis* also indicated that the BR biosynthetic mutant *det2* accumulates more anthocyanins than the wild type [116,120], indicating that BR acts as a negative regulator of anthocyanin biosynthesis. BR may cross talk with JA and CK signals to regulate anthocyanin biosynthesis [121,122]. It was indicated that JA-induced anthocyanin accumulation was repressed in BR mutants or the wild type treated with brassinazole, which is an inhibitor of BR biosynthesis, whereas it was induced via an application of exogenous BR. Further study showed that BR affects JA-induced anthocyanin accumulation by regulating the LBGs, and this regulation might be mediated by the WD-repeat/MYB/bHLH transcriptional complex [121].

### 3.6. Cytokinin Mediates Anthocyanin Biosynthesis

Cytokinin (CK) is an important plant hormone that controls plant organ formation, seed germination, senescence, etc. [123]. There was a study that showed that exogenous CK treatment promotes *Arabidopsis* accumulating more anthocyanin pigments [124], which indicates that CK may play a positive role in regulating plant anthocyanin biosynthesis. CK enhances sucrose-mediated anthocyanin pigmentation, and the CK sensors (AHK2/3/4), histidine-containing phosphotransfer proteins (AHP2/3/5), and master TFs (type B ARR1/10/12) in CK signaling mediate this process in *Arabidopsis* [1,125] (Figure 3). However, CK seems to play a negative role during the high-salinity-induced anthocyanin accumulation. It is reported that the CK-signaling-defective mutants *ahp2*,*3*,*5* and *arr1,10,12* triple mutants show more anthocyanin accumulations after a high-salinity treatment [126]. This indicates that CK-mediated anthocyanin biosynthesis is very complex and CK may cross talk with other signals to regulate anthocyanin biosynthesis and cope with the change in environment, such as high salinity. And further investigation should focus on the mechanism of how CK interacts with other signals to regulate anthocyanin biosynthesis under a stress environment.

### 3.7. Ethylene and Anthocyanin Biosynthesis

Ethylene participates in many biological processes, such as plant growth, senescence, fruit ripening, and stress responses [127,128]. There are also reports showing that ethylene negatively regulates anthocyanin pigmentation in many plants [47,129,130]. It was found that ethylene inhibited sucrose- and light-induced-anthocyanin accumulation in *Arabidopsis* [47,129,131]. The mutants of key components in *Arabidopsis* ethylene signaling, such as *etr1-1*, *ein2-1*, and *ein3 eil1*, all showed ethylene-insensitive and enhanced anthocyanin accumulation phenotypes and further investigation showed that ethylene represses anthocyanin biosynthesis by upregulating the expression of the negative TF *MYBL2* while downregulating the expression of positive TFs, such as *MYB75*, *GL3*, and *TT8* [47] (Figure 3). In tomato, exogenous ethylene treatment significantly repressed anthocyanin accumulation and the expression of *SlAN2-like* and other anthocyanin-related genes [130]. A recent study indicates an ethylene-responsive transcription factor PpERF9 inhibits anthocyanin biosynthesis through epigenetic repression of *PpRAP2.4* and *PpMYB114* via histone deacetylation in pear [50] (Figure 3). However, ethylene can promote anthocyanin accumulation in certain fruits by upregulating genes related to anthocyanin synthesis or increasing the activity of enzymes involved in anthocyanin metabolism. This was observed in fruits such as plum (*Prunus* spp.), grape (*Vitis vinifera* L.), and strawberry (*Fragaria* × *ananassa*) [132,133,134]. Furthermore, when dark-grown sorghum seedlings, which were treated with ethylene, were subsequently exposed to light, the anthocyanin levels increased compared with those without treatment [131]. In summary, the relationship between ethylene and anthocyanin is complex and warrants further exploration in future studies.

### 3.8. GA Negatively Regulates Anthocyanin Biosynthesis

GA is one of the important plant hormones that regulate a diverse range of processes associated with plant growth and development [135]. There are many studies that showed that GA acts as a negative regulator in plant anthocyanin biosynthesis [37]. For instance, a study showed that exogenous application of GA treatment could significantly reduce anthocyanin accumulation in *Arabidopsis* wild-type seedlings [38]. GA also negatively regulates low-temperature-induced anthocyanin accumulation in a HY5/HYH-dependent manner [136]. Zhang et al. 2017 found that a DELLA protein, namely, RGA, can strongly interact with PAP1/MYB75 and enhance its transcriptional activity in *Arabidopsis*, thereby leading to the upregulation of the LBGs under a nitrogen deficiency condition [37]. GA signaling may engage in cross-talk with ABA and JA signaling-mediated anthocyanin biosynthesis, where it was shown that DELLA proteins can promote anthocyanin biosynthesis through sequestering MYBL2 and JAZ suppressors of the MBW complex in *Arabidopsis* [38] (Figure 3).

### 3.9. Auxin and Anthocyanin Biosynthesis

Auxin is an important phytohormone that governs plant growth, development, and responses to environmental variations [137,138,139]. Early studies found that exogenous indole acetic acid (IAA) represses *Sorghum* and *Brassica* anthocyanin accumulation in a dose-dependent manner [140,141], indicating that auxin may play a negative role in regulating anthocyanin biosynthesis in plants. Later studies in many plants confirmed that high auxin can repress plant anthocyanin accumulation [51,52,142,143]. Recently, studies in apple showed that auxin inhibits anthocyanin biosynthesis through the Aux/IAA-ARF signaling pathway [51]. On one hand, a TF MdARF13 binds the promoter of *MdDFR* and inhibits its transcription; on the other hand, MdARF13 also destabilizes the MBW complex by competitively interacting with MdMYB10, which is a key member of the MBW complex in apple. When the auxin level is low, the auxin/indole-3-acetic acid (Aux/IAA) repressor MdIAA121 binds MdARF13 and restrains it from directly binding the *MdDFR* promoter or interacting with MdMYB10 [51] (Figure 3). Furthermore, another study in apple also found that MdIAA26 acts as a positive regulator that promotes anthocyanin accumulation, while auxin promotes the degradation of MdIAA26. However, further investigation is still needed to determine which MdARF is the target of MdIAA26 [52]. Recently, there was a study in sweet cherry that showed that the synthetic auxin 1-naphthaleneacetic acid (NAA) treatment enhances the anthocyanin pigments during the straw-color stage of fruit development, probably by regulating ethylene and ABA metabolism [144]. This indicates that auxin may cross talk with other signals to regulate anthocyanin biosynthesis, and further studies should focus on the mechanisms of how auxin interacts with other signals to control anthocyanin homeostasis in plants.

## 4. Unveiling the Future: Anthocyanins’ Revolutionary Role in Agriculture, Food, and Horticulture

Anthocyanins, which comprise a class of natural pigments responsible for the vibrant hues of various fruits, vegetables, and flowers, have gained significant attention due to their potential health benefits [145,146]. As scientific studies continue to unravel the multifaceted properties of anthocyanins, their applications in agriculture and food processing are emerging as promising avenues for enhancing visual appeal, nutritional content, and overall consumer satisfaction.

### 4.1. Crop Color Enhancement

**Figure 4:**
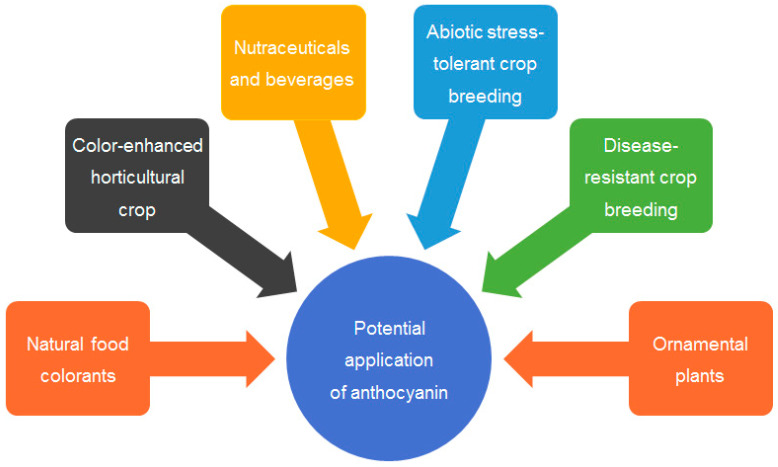
Anthocyanins’ potential applications in agriculture, food, and horticulture. Anthocyanins have various potential applications in food, crops, and horticulture due to their health benefits, enhanced stress tolerance, and bright colors.

Synthetic food colorants have raised concerns regarding their safety and impact on health. Anthocyanins offer a natural alternative to food coloring, enabling food processors to meet consumer demand for visually appealing and safe products without compromising health [147]. Anthocyanin-rich foods not only add vibrant colors but also contribute to nutritional enhancement. Incorporating these pigments into a variety of processed foods can elevate their antioxidant and phytonutrient contents, thereby improving the overall nutritional profile. Anthocyanin-rich crops have the potential to revolutionize the esthetics of agricultural landscapes. Manipulating anthocyanin biosynthesis through genetic engineering or selective breeding can result in visually appealing crops, thereby increasing consumer interest and market value (Figure 4).

### 4.2. Stress-Tolerant Crop Breeding

Anthocyanins have been linked to enhanced stress tolerance in plants, including resistance to various abiotic stresses and biotic stresses [2,4,93]. Incorporating these traits into crops can lead to improved resilience, increased yield stability, and sustainable agricultural practices, which may provide significant guidance to crop breeding and improvement (Figure 4).

### 4.3. Ornamental Plant Innovations

The utilization of anthocyanins can extend beyond edibles to ornamental plants [148]. Developing new cultivars with vibrant colors and prolonged bloom periods can significantly enhance the ornamental horticulture industry. Furthermore, anthocyanin-rich plants can contribute to urban greening initiatives to beautify cityscapes while also providing ecosystem services, such as air purification and temperature regulation (Figure 4).

The growing body of research on anthocyanins’ health benefits and diverse applications has sparked interest across the agricultural and food industries. From enhancing crop aesthetics to improving nutritional content and contributing to sustainable agricultural practices, anthocyanins hold immense promise. As scientific knowledge advances and consumer preferences shift toward natural and healthier options, the integration of anthocyanins in agriculture, food processing, and horticulture is poised to play a pivotal role in shaping the future of these industries. Thus, identifying additional regulatory and structural genes controlling anthocyanin biosynthesis in various plants and deciphering their regulatory networks holds significant scientific importance for the future molecular breeding of crops and horticultural species.

## Figures and Tables

**Table 1:** List of genes related to MBW complex and anthocyanin accumulation in plants.

Protein	Plant Species	Environmental Stimuli and Phytohormones	Function Regarding Anthocyanin	References
HY5	*Arabidopsis* (Col-0, Ler, No-0)	Light	Upregulation	[17]
COP1	*Arabidopsis* (Col-0)	Light	Downregulation	[27]
VvBBX44	Grape (*Vitis vinifera* L.)	Light	Downregulation	[28]
MYB112	*Arabidopsis* (Col-0)	Light and salinity	Upregulation	[29]
HAT1	*Arabidopsis* (Col-0)	Light	Downregulation	[30]
TPL	*Arabidopsis* (Col-0)	Light	Downregulation	[30]
JAZ1/6/8/11	*Arabidopsis* (Col-0)	Jasmonate	Downregulation	[31,32]
ECAP	*Arabidopsis* (Col-0)	Jasmonate, salinity, and drought	Downregulation	[31,33]
MdMYB308L	Apple (*Malus domestica*)	Cold	Upregulation	[34]
NLA	*Arabidopsis* (Col-0)	Low nitrogen	Upregulation	[35]
DELLA	*Arabidopsis* (Col-0, Ler)	Gibberellin, low phosphorus, and low nitrogen	Upregulation	[36,37,38]
SPX4	*Arabidopsis* (Col-0)	Low phosphorus	Downregulation	[39]
PHR1	*Arabidopsis* (Col-0)	Low phosphorus	Upregulation	[40]
SMXL6/7/8	*Arabidopsis* (Col-0)	Strigolactone	Downregulation	[41]
COI1	*Arabidopsis* (Col-0)	Jasmonate	Upregulation	[42]
MdMYB9	Apple (*Malus domestica*)	Jasmonate	Upregulation	[43]
MdTRB1	Apple (*Malus domestica*)	Jasmonate	Upregulation	[43]
MdJAZ1	Apple (*Malus domestica*)	Jasmonate	Downregulation	[43]
VvSPL9	Grape (*Vitis vinifera* L.)	Jasmonate	Downregulation	[44]
MdABI5	Apple (*Malus domestica*)	Abscisic acid	Upregulation	[45]
MYBL2	*Arabidopsis* (Col-0)	Ethylene, Cytokinin and Abscisic acid	Downregulation	[46,47]
Elongator	*Arabidopsis* (Col-0)	Abscisic acid	Downregulation	[46]
NPR1	*Arabidopsis* (Col-0)	Salicylic acid	Upregulation	[48]
MdJa2	Apple (*Malus domestica*)	Brassinosteroid	Downregulation	[49]
EIN2	*Arabidopsis* (Col-0, WS)	Ethylene	Downregulation	[47]
EIN3/EIL1	*Arabidopsis* (Col-0, WS)	Ethylene	Downregulation	[47]
ETR1	*Arabidopsis* (Col-0, WS)	Ethylene	Downregulation	[47]
PpERF9	Pear (*Pyrus* spp.)	Ethylene	Downregulation	[50]
MdARF13	Apple (*Malus domestica*)	Auxin	Downregulation	[51]
MdIAA26	Apple (*Malus domestica*)	Auxin	Upregulation	[52]

## Data Availability

Not applicable.
